# Do worse baseline risk factors explain the association of healthy obesity with increased mortality risk? Whitehall II Study

**DOI:** 10.1038/s41366-018-0192-0

**Published:** 2018-08-14

**Authors:** William Johnson, Joshua A. Bell, Ellie Robson, Tom Norris, Mika Kivimäki, Mark Hamer

**Affiliations:** 1 School of Sport, Exercise and Health Sciences, Loughborough University, Loughborough, UK; 2 MRC Integrative Epidemiology Unit at the University of Bristol, Bristol, UK; 3 Department of Health Sciences, University of Leicester, Leicester, UK; 4 Department of Epidemiology and Public Health, University College London, London, UK

**Keywords:** Obesity, Epidemiology

## Abstract

**Objective:**

To describe 20-year risk factor trajectories according to initial weight/health status and investigate the extent to which baseline differences explain greater mortality among metabolically healthy obese (MHO) individuals than healthy non-obese individuals.

**Methods:**

The sample comprised 6529 participants in the Whitehall II study who were measured serially between 1991–1994 and 2012–2013. Baseline weight (non-obese or obese; body mass index (BMI) ≥30 kg/m^2^) and health status (healthy or unhealthy; two or more of hypertension, low high-density lipoprotein cholesterol (HDL-C), high triglycerides, high glucose, and high homeostatic model assessment of insulin resistance (HOMA-IR)) were defined. The relationships of baseline weight/health status with 20-year trajectories summarizing ~25,000 observations of systolic and diastolic blood pressures, HDL-C, triglycerides, glucose, and HOMA-IR were investigated using multilevel models. Relationships of baseline weight/health status with all-cause mortality up until July 2015 were investigated using Cox proportional hazards regression.

**Results:**

Trajectories tended to be consistently worse for the MHO group compared to the healthy non-obese group (e.g., glucose by 0.21 (95% CI 0.09, 0.33; *p* < 0.001) mmol/L at 20-years of follow-up). Consequently, the MHO group had a greater risk of mortality (hazard ratio 2.11 (1.24, 3.58; *p* = 0.006)) when the referent group comprised a random sample of healthy non-obese individuals. This estimate, however, attenuated (1.34 (0.85, 2.13; *p* = 0.209)) when the referent group was matched to the MHO group on baseline risk factors.

**Conclusions:**

Worse baseline risk factors may explain any difference in mortality risk between obese and non-obese groups both labelled as healthy, further challenging the concept of MHO.

## Introduction

Obesity is a major public health problem because of its adverse consequences for long-term health and well-being [1]. Clearly, however, not all obese individuals have the same cardio-metabolic disease risk factor (e.g., blood pressure and fasting glucose levels) profiles. The concept that someone can be obese yet metabolically healthy, most commonly called “metabolically healthy obesity (MHO)”, has been highly controversial and widely debated [2, 3]. In particular, two main types of epidemiological studies have questioned whether or not MHO is truly a benign condition, relative to metabolically healthy non-obese (MHNO). The first has investigated the progression of the MHO phenotype over time, demonstrating that this group tends to develop risk factors and transition to being unhealthy more frequently than their non-obese counterparts [4–11]. Hamer et al. [10] for example, demonstrated that, over 8 years of follow-up in the English Longitudinal Study of Ageing, 45% of MHO participants transitioned to an unhealthy state compared to 17% of MHNO participants [10]. The second type of study has investigated disease prognosis or mortality, demonstrating a ranking of risk according to both weight and health status [12–20]. Lassale et al. [17] for example, have recently reported hazard ratios for incident coronary heart disease, among ~18,000 European adults, of 1.28 (95% confidence interval (CI) 1.03, 1.58) for MHO, 2.15 (1.79, 2.57) for metabolically unhealthy non-obese (MUNO), and 2.54 (2.21, 2.92) for metabolically unhealthy obesity (MUO), compared to a MHNO referent group [17]. Existing studies can therefore be summarised as providing evidence that MHO is an intermediate state before the development of cardio-metabolic abnormalities and, as such, is related to increased disease and mortality risk.

A notable limitation of this literature is that all definitions of MHO, most of which are based on blood pressure, high-density lipoprotein cholesterol (HDL-C), triglycerides, and plasma glucose, require continuous variables to be dichotomised (e.g., blood pressures to hypertension status). This process often results in systematic differences in baseline descriptive statistics among healthy or unhealthy individuals, according to weight status [4–18]. For example, the Lassale et al. publication reported baseline levels of triglycerides of 1.13 (1.10, 1.15) mmol/L in the MHNO group and 1.22 (1.16, 1.27) in the MHO group [17]. These inherent differences appear to be relatively small, but we do not know how they change over follow-up, because no study has described trajectories of continuous risk factors over time according to baseline weight/health status. Such trajectories would help understand why so-called MHO individuals often transition to being unhealthy. Moreover, previous studies have largely failed to consider the extent to which inherent baseline differences might explain the observed greater disease and mortality rates of obese compared to non-obese individuals, who are all apparently healthy. Evidence that both groups have the same mortality risk when they are matched on baseline cardio-metabolic disease risk factors would further challenge the concept of MHO.

Using longitudinal data from the Whitehall II study, we aimed to describe 20-year risk factor trajectories according to initial weight/health status and investigate the extent to which baseline differences might explain the expected greater mortality of MHO compared to MHNO individuals.

## Subjects and methods

### Study sample

The Whitehall II study was established to explore the relationship between socio-economic position, stress and cardiovascular disease [21]. A cohort of 10,308 (6895 men; 3413 women) civil servants aged 35–55 years, working in London, United Kingdom (UK) for the government, participated in the baseline examination in 1985–1988 (response 74%). A combination of clinical and questionnaire data from five repeated assessments (1991–1994, 1997–1999, 2002–2004, 2007–2009, and 2012–2013) over approximately two decades of follow-up were used in the present paper. The 1991–1994 assessment was the first to cover a large range of biological measurements, including fasting glucose, and was used as baseline in this analysis. The University College London research ethics committee granted ethical approval for each phase of data collection. Participants provided written informed consent.

Starting with the 8815 cohort members who were still participating in the study at baseline in 1991/1994, 1769 were excluded because of missing clinical data necessary to define weight/health status at baseline, a further 50 because their body mass index (BMI) at baseline was classified as thin (i.e., <18.5 kg/m^2^), 457 because of missing baseline covariate data, and 10 because they did not have a single measurement of waist circumference across the five assessments. The resulting sample comprised 6529 (4604 men; 1925 women) individuals, representing 71% of the eligible cohort (i.e., *N* = 8815) and 63% of the total cohort (i.e., *N* = 10,308).

### Data

#### Clinical measurements

At each assessment, weight, height, and waist circumference (at the widest point) were measured by a trained nurse according to standardized protocols, systolic blood pressure (SBP) and diastolic blood pressure (DBP) were assessed, and fasting blood samples were taken for biochemical analysis of HDL-C, triglycerides, glucose, and insulin levels, as previously described [5, 6, 22, 16, 21]. BMI was calculated as weight (kg)/height (m)^2^ and homeostatic model assessment of insulin resistance (HOMA-IR) as fasting glucose (mmol/L) × fasting insulin (mmol/L)/22.5.

In total, there were 24,903 observations of BMI, 24,318 of waist circumference, 25,608 of SBP, 25,607 of DBP, 24,736 of HDL-C, 25,317 of triglycerides, 25,282 of fasting glucose, and 23,805 of HOMA-IR. For each of these outcomes, ~70% of the sample had four or five observations and ~65% of the sample was followed-up for more than 17.5 years.

#### Covariates

Covariates at baseline were assessed via a questionnaire and coded as follows. Age in decimal years (centred about the mean), sex (female vs male), and ethnicity (non-white vs white) were recorded in addition to socio-economic position based on occupational role (clerical/support, administrative, vs professional/executive). The following health behaviours were also assessed: frequency of alcohol consumption (daily, weekly or monthly, vs never or special occasions), smoking status (current, ex, vs never), and frequency of mild and moderate exercise (1–3 times/month or seldom, 1–2 times/week, vs 3 times/week or more). The General Health Questionnaire (GHQ-30) was administered to capture psychological distress and was coded as 10–30, 2–9, vs 0–1 after inspecting the distribution to create three reasonably sized categories [23]. Finally, diet was assessed via questions on the frequency of consumption of 10 fruits and 18 vegetables, bread (white, brown, and wholemeal), and milk (whole, semi-skimmed, skimmed, and others). Fruit and vegetable consumption were defined as healthy if they reported eating any item once a day or more, unhealthy if they did not eat any item at least 2–4 times a week, and moderately healthy for everything in-between. Bread consumption was defined as healthy if they reported eating brown bread or wholemeal bread more frequently than white bread, unhealthy if they ate white bread most frequently, and moderately healthy for everything else. Milk consumption was defined as healthy if they did not use any milk or used skimmed or other types of milk, unhealthy if they used whole milk, and moderately healthy if they used semi-skimmed milk. A slightly modified version of a composite dietary pattern score, used in previous Whitehall II study publications, was then created [24]. Individuals were defined as healthy if they were healthy on all sub-scales (i.e., fruit and vegetable, bread, and milk) with the allowance of being moderately healthy on one sub-scale, unhealthy if they were unhealthy on all sub-scales with the allowance of being moderately healthy on one sub-scale, and everyone else was classified as moderately healthy.

At each assessment, use of hypertension medication (diuretics, beta-blockers, ACE inhibitors, calcium channel blockers, and other antihypertensives), diabetes medication (insulin and oral antidiabetic drugs), and cardiovascular disease medication (antihypertensives, nitrates, antiplatelets, and lipid-lowering drugs) medication were reported (yes vs no).

#### Mortality records

Individual participant data were linked to death records from the National Health Service (NHS) Central Register, using NHS identification numbers, up until July 2015. Six participants were missing these data and were excluded from analyses of all-cause mortality.

### Statistical analyses

Weight status at baseline was defined as non-obese (18.5–29.9 kg/m^2^) or obese (≥30.0 kg/m^2^). On the basis of independent criteria [25], and as in previous Whitehall II publications [5, 6], health status at baseline was defined as healthy if participants had zero or one of the following five cardio-metabolic disease risk factors and unhealthy if they had two or more: blood pressure ≥130/85 mmHg or use of hypertension medication, HDL-C <1.03 mmol/L for men and <1.29 mmol/L for women, triglycerides ≥1.7 mmol/L, fasting plasma glucose ≥5.6 mmol/L or use of diabetes medication, and HOMA-IR >3.17 (90th percentile in the sample). Individuals were then categorised as being MHNO, MHO, MUNO, or MUO at baseline.

Descriptive statistics for baseline variables were produced stratified according to weight/health status, and between-group differences (MHO vs MHNO and MUO vs MUNO) were tested using *χ*^2^-tests for categorical variables and *t*-tests for continuous variables.

#### Trajectory modelling

Trajectories were modelled in a multilevel general linear regression framework (measurement occasion at level one and individuals at level two) [26, 27], incorporating systematic differences in the sample-average trajectory according to baseline weight/health status and adjustment for covariates.

A separate model was built for each of the eight outcomes (i.e., serial BMI, waist circumference, SBP, DBP, HDL-C, triglycerides, glucose, and HOMA-IR). HDL-C, triglycerides, glucose, and HOMA-IR were log transformed due to skewed distributions, but all presented results have been back-transformed to the original scales. The time scale was decimal years of follow-up, modelled as a quadratic polynomial function to allow nonlinear trajectories. Exploratory analyses revealed that more complex functions, including fractional polynomials and restricted cubic splines, did not result in better fitting models or noticeably different sample-average trajectories. The constant and quadratic polynomial terms (i.e., time and time^2^) were allowed to have random effects at level two, with an unstructured variance-covariance matrix.

In all instances, the baseline weight/health status exposure was included as a main effect and as an interaction with the quadratic polynomial terms, thereby allowing the sample-average trajectory to be truly different for each group. Adjustment was made for sex, ethnicity, baseline covariates (age, alcohol, smoking, mild and moderate exercise, occupational grade, GHQ, and diet) and medication at each assessment (hypertension medication for SBP and DBP, cardiovascular disease medication for HDL-C and triglycerides, and diabetes medication for glucose and HOMA-IR).

The resulting covariate-adjusted trajectories, according to weight/health status at baseline, were plotted for each outcome separately. The models were also used to estimate mean (95% CI) values and contrast differences between groups (MHO vs MHNO and MUO vs MUNO) at baseline and at 20 years of follow-up.

#### Survival analysis

To illustrate how even baseline differences in cardio-metabolic disease risk factors may explain differences in subsequent mortality between MHO and MHNO groups, survival analysis was applied to three different samples. The first “full sample” comprised all MHO and MHNO individuals, the second “random sample” comprised the 270 MHO individuals (from the full sample) plus 270 randomly selected MHNO individuals, and the third “matched sample” comprised the 270 MHO individuals plus 270 MHNO individuals who were matched based on SBP and DBP and log-transformed HDL-C, triglycerides, glucose, and HOMA-IR at baseline. Propensity score matching was implemented using logistic regression without replacement, so that the matched group did not include the same individual more than once. Matching was based on the best propensity score for each MHNO individual and not any other criteria (e.g., SBP within 2 mmHg etc.). To assess the quality of the matching, differences in cardio-metabolic disease risk factors at baseline between the MHNO and MHO groups were estimated within each sample using general linear regression models. HDL-C, triglycerides, glucose, and HOMA-IR were log-transformed due to skewed distributions; regression estimates were exponentiated and can be interpreted as ratios of geometric means.

For each sample, a Cox proportional hazards regression model was built to test the association of weight/health status (MHO vs MHNO) with all-cause mortality. Age at death was recorded and decimal years were the time scale for follow-up. For participants with no record of an event, the data were censored at July 2015. Adjustment was made for sex, ethnicity, and baseline covariates (age, alcohol, smoking, mild and moderate exercise, occupational grade, GHQ, and diet). After fitting each model, the proportional hazards assumption was examined using log–log plots and tested using Schoenfeld residuals (all *p*-values >0.3, indicating no violation).

All procedures were performed in Stata 15 (StataCorp LP, College Station, TX, USA). The command runmlwin was used for the multilevel models [28].

#### Code availability

The statistical code for the analyses in this paper is available upon request from the corresponding author.

## Results

Table 1 shows descriptive statistics of the study sample at baseline, according to weight/health status. Approximately 90% of the sample was non-obese and 75% of these individuals were healthy. Conversely, among the 10% of the sample that was obese only 40% were healthy. Despite both the MHNO and MHO groups being labelled as healthy, average levels of all cardio-metabolic disease risk factors (except for glucose) were worse in the MHO group (e.g., SBP 122.4 vs 117.7 mmHg; HOMA-IR 1.69 vs 1.03). Similarly, average levels of most cardio-metabolic disease risk factors were worse in the MUO group compared to the MUNO group.

**Table 1:** Description of study sample at baseline, according to weight/health status

		MHNO (*N* = 4371)	MHO (*N* = 272)	*P*-value	MUNO (*N* = 1487)	MUO (*N* = 399)	*P*-value
*Sex*				<0.001			<0.001
Male	*N* (%)	3006 (69)	106 (39)		1256 (84)	236 (59)	
Female	*N* (%)	1365 (31)	166 (61)		231 (16)	163 (41)	
*Ethnicity*				<0.001			0.668
White	*N* (%)	4044 (93)	229 (84)		1300 (87)	352 (88)	
Non-white	*N* (%)	327 (7)	43 (16)		187 (13)	47 (12)	
Age (years)	Mean (SD)	49.7 (6.0)	50.5 (5.8)	0.039	51.2 (6.1)	51.0 (6.0)	0.504
BMI (kg/m^2^)	Mean (SD)	24.2 (2.5)	32.5 (2.6)	<0.001	25.9 (2.2)	33.6 (3.6)	<0.001
WC (cm)	Mean (SD)	82.8 (9.7)	98.5 (10.5)	<0.001	90.3 (8.2)	105.3 (9.6)	<0.001
SBP (mmHg)	Mean (SD)	117.7 (12.0)	122.4 (12.8)	<0.001	127.9 (14.5)	131.1 (13.2)	<0.001
DBP (mmHg)	Mean (SD)	77.8 (8.4)	81.6 (8.9)	<0.001	84.8 (9.3)	87.1 (9.1)	<0.001
HDL-C (mmol/L)	Median (IQR)	1.47 (1.24, 1.74)	1.42 (1.24, 1.64)	0.046	1.07 (0.92, 1.33)	1.12 (0.94, 1.34)	0.629
Triglycerides (mmol/L)	Median (IQR)	1.02 (0.77, 1.37)	1.17 (0.95, 1.53)	<0.001	2.04 (1.59, 2.70)	2.05 (1.52, 2.82)	0.457
Glucose (mmol/L)	Median (IQR)	5.10 (4.90, 5.40)	5.10 (4.80, 5.30)	0.087	5.50 (5.20, 5.80)	5.60 (5.20, 6.00)	0.020
HOMA-IR	Median (IQR)	1.03 (0.66, 1.53)	1.69 (1.19, 2.29)	<0.001	1.91 (1.22, 3.12)	3.44 (2.34, 4.85)	<0.001
*Alcohol*				<0.001			<0.001
Never or special occasions	*N* (%)	631 (14)	73 (27)		229 (15)	106 (27)	
Weekly or monthly	*N* (%)	2220 (51)	119 (44)		722 (49)	185 (46)	
Daily	*N* (%)	1520 (35)	80 (29)		536 (36)	108 (27)	
*Smoking*				0.783			0.060
Never	*N* (%)	2143 (49)	128 (47)		664 (45)	152 (38)	
Ex	*N* (%)	1646 (38)	108 (40)		604 (41)	184 (46)	
Current	*N* (%)	582 (13)	36 (13)		219 (15)	63 (16)	
*Mild exercise*				<0.001			<0.001
Three times/week or more	*N* (%)	2977 (68)	164 (60)		970 (65)	217 (54)	
1–2 times/week	*N* (%)	1101 (25)	73 (27)		390 (26)	122 (31)	
1–3 times/month or seldom	*N* (%)	293 (7)	35 (13)		127 (9)	60 (15)	
*Moderate exercise*				<0.001			<0.001
Three times/week or more	*N* (%)	714 (16)	32 (12)		215 (15)	32 (8)	
1–2 times/week	*N* (%)	1990 (46)	94 (34)		712 (48)	159 (40)	
1–3 times/month or seldom	*N* (%)	1667 (38)	146 (54)		560 (38)	208 (52)	
*GHQ*				0.729			0.005
0–1	*N* (%)	2657 (61)	169 (62)		983 (66)	230 (58)	
2–9	*N* (%)	1234 (28)	71 (26)		384 (26)	124 (31)	
10–30	*N* (%)	480 (11)	32 (12)		120 (8)	45 (11)	
*Grade*				<0.001			<0.001
Professional or executive	*N* (%)	1975 (45)	134 (49)		697 (47)	173 (43)	
Administrative	*N* (%)	1731 (40)	69 (25)		582 (39)	115 (29)	
Clerical or support	*N* (%)	665 (15)	69 (25)		208 (14)	111 (28)	
*Diet*				0.816			0.321
Healthy	*N* (%)	1299 (30)	82 (30)		374 (25)	115 (29)	
Moderately healthy	*N* (%)	2516 (58)	159 (58)		858 (58)	221 (55)	
Unhealthy	*N* (%)	556 (13)	31 (11)		255 (17)	63 (16)	

*P*-values are from *χ*^2^-tests for categorical variables and *t*-tests for continuous variables (HDL-C, triglycerides, glucose, and HOMA-IR were log-transformed due to skewed distributions)

*BMI* body mass index, *DBP* diastolic blood pressure, *GHQ* general health questionnaire, *HDL-C* high-density lipoprotein cholesterol, *HOMA-IR* homeostatic model assessment of insulin resistance, *MHNO* metabolically healthy non-obese, *MHO* metabolically healthy obese, *MUNO* metabolically unhealthy non-obese, *MUO* metabolically unhealthy obese, *SBP* systolic blood pressure, *WC* waist circumference

### Trajectories

Overall fits of the multilevel trajectory models were determined to be good according the residual standard deviations, a measure of average error on the original scale used for modelling (e.g., BMI 1.1 kg/m^2^ and DBP 6.5 mmHg). Further, diagnostic plots of the level-one random effects indicated comparable fit across the four weight/health groups and that the trajectories were not systematically too high or low across time (data not shown).

**Fig. 1:**
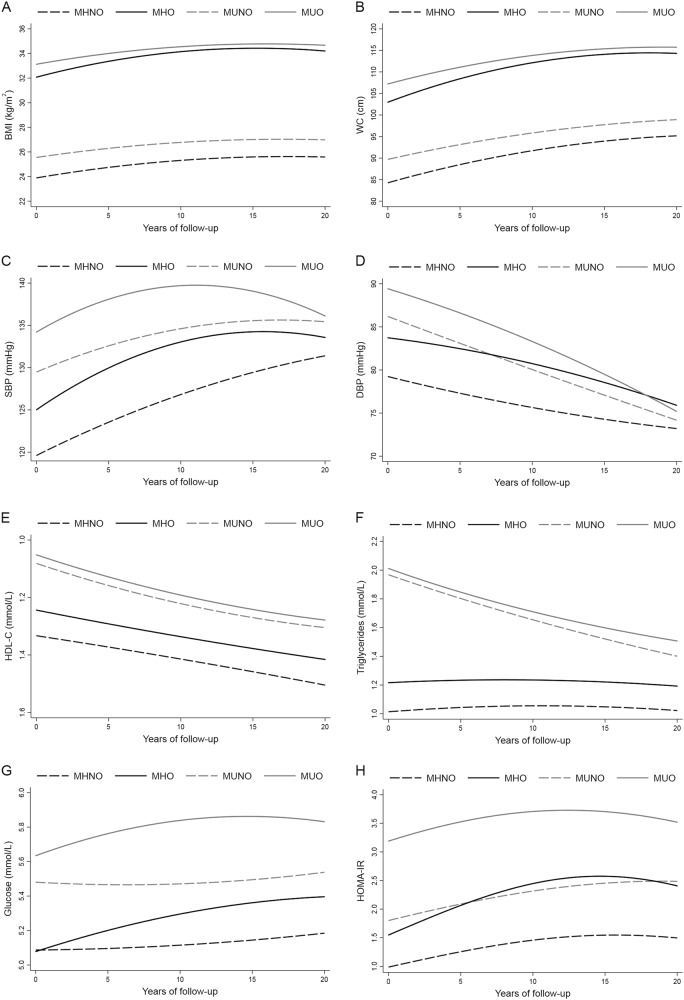
Trajectories of risk factors over 20 years of follow-up according to weight/health status at baseline, estimated using multilevel models. The multilevel models were adjusted for sex, ethnicity, baseline covariates (age, alcohol, smoking, mild and moderate exercise, occupational grade, GHQ, and diet), and medication at each assessment (for SBP, DBP, HDL-C, triglycerides, glucose, and HOMA-IR outcomes). MHNO *N* = 4371, MHO *N* = 272, MUNO *N* = 1487, MUO *N* = 399. *BMI* body mass index, *DBP* diastolic blood pressure, *GHQ* general health questionnaire, *HDL-C* high-density lipoprotein cholesterol, *HOMA-IR* homeostatic model assessment of insulin resistance, *MHNO* metabolically healthy non-obese, *MHO* metabolically healthy obese, *MUNO* metabolically unhealthy non-obese, *MUO* metabolically unhealthy obese, *SBP* systolic blood pressure, *WC* waist circumference

Figure 1 shows the estimated covariate-adjusted trajectories for each of the outcomes, according to weight/health status at baseline. The *y*-axis for HDL-C is reversed so that higher trajectories indicate worse levels for all outcomes. For BMI and WC (Panels A & B), trajectories were consistently higher for (1) the MUO group compared to the MHO group and (2) the MUNO group compared to the MHNO group. For the cardio-metabolic disease risk factors (Panels C–H), trajectories tended to be higher for the MHO group compared to the MHNO group; these differences had *p*-values <0.05 at baseline and at 20 years of follow-up, with the exceptions of glucose at baseline and SBP at 20 years of follow-up (Table 2). Further, cardio-metabolic disease risk factor trajectories tended to be higher for the MUO group compared to the MUNO group, although after 20 years of follow-up these differences were less apparent for SBP, DBP, and HDL-C (Table 3).

**Table 2:** Estimated values of risk factors in MHNO and MHO groups from multilevel trajectory models

	MHNO (*N* = 4371)	MHO (*N* = 272)	MHO–MHNO	
	Mean (95% CI)	Mean (95% CI)	Mean (95% CI)	*P*-value
*Baseline*				
BMI (kg/m^2^)	23.9 (23.6, 24.2)	32.1 (31.7, 32.5)	8.2 (7.9, 8.5)	<0.001
WC (cm)	84.3 (83.4, 85.1)	103.0 (101.7, 104.2)	18.7 (17.7, 19.7)	<0.001
SBP (mmHg)	119.6 (118.3, 120.9)	125.0 (122.9, 127.1)	5.4 (3.7, 7.1)	<0.001
DBP (mmHg)	79.2 (78.4, 80.1)	83.7 (82.4, 85.1)	4.5 (3.3, 5.7)	<0.001
HDL-C (mmol/L)	1.33 (1.30, 1.36)	1.22 (1.18, 1.26)	−0.11 (−0.15, −0.08)	<0.001
Triglycerides (mmol/L)	1.01 (0.97, 1.06)	1.22 (1.14, 1.30)	0.20 (0.14, 0.27)	<0.001
Glucose (mmol/L)	5.09 (5.04, 5.14)	5.08 (5.00, 5.16)	−0.01 (−0.08, 0.06)	0.806
HOMA-IR	0.99 (0.93, 1.04)	1.55 (1.41, 1.68)	0.56 (0.44, 0.68)	<0.001
*20 years follow-up*				
BMI (kg/m^2^)	25.6 (25.3, 25.9)	34.2 (33.6, 34.8)	8.6 (8.1, 9.1)	<0.001
WC (cm)	95.2 (94.3, 96.0)	114.3 (112.7, 115.9)	19.1 (17.7, 20.6)	<0.001
SBP (mmHg)	131.4 (130.0, 132.8)	133.6 (130.9, 136.2)	2.2 (−0.2, 4.6)	0.078
DBP (mmHg)	73.2 (72.3, 74.1)	75.9 (74.2, 77.65)	2.7 (1.2, 4.2)	<0.001
HDL-C (mmol/L)	1.51 (1.47, 1.54)	1.38 (1.32, 1.43)	−0.13 (−0.18, −0.08)	<0.001
Triglycerides (mmol/L)	1.02 (0.98, 1.07)	1.19 (1.11, 1.28)	0.17 (0.10, 0.24)	<0.001
Glucose (mmol/L)	5.18 (5.13, 5.24)	5.40 (5.27, 5.52)	0.21 (0.09, 0.33)	0.001
HOMA-IR	1.48 (1.40, 1.57)	2.39 (2.13, 2.65)	0.91 (0.67, 1.14)	<0.001

The multilevel models were adjusted for sex, ethnicity, baseline covariates (age, alcohol, smoking, mild and moderate exercise, occupational grade, GHQ, and diet), and medication at each assessment (for SBP, DBP, HDL-C, triglycerides, glucose, and HOMA-IR outcomes). The estimates correspond to the trajectories shown in Fig. 1

*BMI* body mass index, *DBP* diastolic blood pressure, *GHQ* general health questionnaire, *HDL-C* high-density lipoprotein cholesterol, *HOMA-IR* homeostatic model assessment of insulin resistance, *MHNO* metabolically healthy non-obese, *MHO* metabolically healthy obese, *MUNO* metabolically unhealthy non-obese, *MUO* metabolically unhealthy obese, *SBP* systolic blood pressure, *WC* waist circumference

**Table 3:** Estimated values of risk factors in MUNO and MUO groups from multilevel trajectory models

	MUNO (*N* = 1487)	MUO (*N* = 399)	MUNO–MUO	
	Mean (95% CI)	Mean (95% CI)	Mean (95% CI)	*P*-value
*Baseline*				
BMI (kg/m^2^)	25.6 (25.3, 25.9)	33.1 (32.7, 33.5)	7.6 (7.3, 7.9)	<0.001
WC (cm)	89.7 (88.8, 90.6)	107.2 (106.1, 108.3)	17.5 (16.6, 18.4)	<0.001
SBP (mmHg)	129.5 (128.0, 130.9)	134.2 (132.3, 136.1)	4.7 (3.2, 6.3)	<0.001
DBP (mmHg)	86.2 (85.3, 87.1)	89.4 (88.2, 90.7)	3.2 (2.2, 4.3)	<0.001
HDL-C (mmol/L)	1.04 (1.01, 1.06)	1.01 (0.97, 1.04)	−0.03 (−0.06, −0.005)	0.020
Triglycerides (mmol/L)	1.97 (1.88, 2.06)	2.01 (1.89, 2.13)	0.04 (−0.06, 0.14)	0.390
Glucose (mmol/L)	5.48 (5.42, 5.54)	5.63 (5.55, 5.72)	0.15 (0.08, 0.22)	<0.001
HOMA-IR	1.80 (1.69, 1.91)	3.19 (2.93, 3.44)	1.39 (1.18, 1.60)	<0.001
*20 years follow-up*				
BMI (kg/m^2^)	27.0 (26.6, 27.3)	34.7 (34.2, 35.2)	7.7 (7.2, 8.2)	<0.001
WC (cm)	98.9 (97.9, 99.9)	115.7 (114.3, 117.2)	16.8 (15.5, 18.2)	<0.001
SBP (mmHg)	135.4 (133.8, 137.1)	136.1 (133.7, 138.5)	0.7 (−1.6, 2.9)	0.550
DBP (mmHg)	74.2 (73.1, 75.2)	75.2 (73.7, 76.7)	1.0 (−0.4, 2.4)	0.153
HDL-C (mmol/L)	1.23 (1.20, 1.26)	1.20 (1.16, 1.24)	−0.03 (−0.07, 0.006)	0.095
Triglycerides (mmol/L)	1.40 (1.33, 1.47)	1.51 (1.41, 1.61)	0.11 (0.02, 0.19)	0.015
Glucose (mmol/L)	5.54 (5.46, 5.61)	5.83 (5.71, 5.95)	0.29 (0.17, 0.41)	<0.001
HOMA-IR	2.44 (2.28, 2.60)	3.36 (3.02, 3.71)	0.92 (0.61, 1.23)	<0.001

The multilevel models were adjusted for sex, ethnicity, baseline covariates (age, alcohol, smoking, mild and moderate exercise, occupational grade, GHQ, and diet), and medication at each assessment (for SBP, DBP, HDL-C, triglycerides, glucose, and HOMA-IR outcomes). The estimates correspond to the trajectories shown in Fig. 1

*BMI* body mass index, *DBP* diastolic blood pressure, *GHQ* general health questionnaire, *HDL-C* high-density lipoprotein cholesterol, *HOMA-IR* homeostatic model assessment of insulin resistance, *MHNO* metabolically healthy non-obese, *MHO* metabolically healthy obese, *MUNO* metabolically unhealthy non-obese, *MUO* metabolically unhealthy obese, *SBP* systolic blood pressure, *WC* waist circumference

### All-cause mortality

Differences in baseline BMI, waist circumference, and cardio-metabolic disease risk factors between MHNO and MHO groups are presented in Table 4 for the full sample, the random sample, and the matched sample. In agreement with the previous results, in the full sample, all risk factors except for glucose were worse in the MHO group compared to the MHNO group. Estimated differences were comparable between the full sample and the random sample. For example, SBP was 4.6 (95% CI 3.1, 6.1) mmHg higher among MHO individuals (than MHNO individuals) in the full sample, compared to 4.8 (2.6, 6.9) mmHg higher among MHO individuals in the random sample. In the matched sample, however, estimated differences between MHNO and MHO groups in all cardio-metabolic disease risk factors were attenuated to the null (e.g., 0.7 (−1.4, 2.8) for SBP), thereby demonstrating that the propensity score matching had served its purpose in creating a referent MHNO group that differed from the MHO group only in BMI and WC.

**Table 4:** Differences in risk factors at baseline between MHNO and MHO groups in the full sample, a 1:1 random sample, and a 1:1 matched sample, estimated using general linear regression models

	Full sample			1:1 random sample			1:1 matched sample		
	MHNO (*N* = 4368)	MHO (*N* = 270)		MHNO (*N* = 270)	MHO (*N* = 270)		MHNO (*N* = 270)	MHO (*N* = 270)	
		B (95% CI)	*P*-value		B (95% CI)	*P*-value		B (95% CI)	*P*-value
BMI (kg/m^2^)	0.0 (ref)	8.3 (8.0, 8.6)	<0.001	0.0 (ref)	8.3 (7.9, 8.8)	<0.001	0.0 (ref)	7.4 (7.0, 7.8)	<0.001
WC (cm)	0.0 (ref)	15.9 (14.7, 17.1)	<0.001	0.0 (ref)	16.1 (14.3, 17.8)	<0.001	0.0 (ref)	12.8 (11.1, 14.5)	<0.001
SBP (mmHg)	0.0 (ref)	4.6 (3.1, 6.1)	<0.001	0.0 (ref)	4.8 (2.6, 6.9)	<0.001	0.0 (ref)	0.7 (−1.4, 2.8)	0.518
DBP (mmHg)	0.0 (ref)	3.7 (2.7, 4.8)	<0.001	0.0 (ref)	4.0 (2.6, 5.5)	<0.001	0.0 (ref)	0.01 (−1.5, 1.5)	0.988
HDL-C (mmol/L)	1.0 (ref)	0.97 (0.94, 1.00)	0.042	1.0 (ref)	0.97 (0.94, 1.01)	0.212	1.0 (ref)	1.00 (0.96, 1.04)	0.868
Triglycerides (mmol/L)	1.0 (ref)	1.17 (1.11, 1.23)	<0.001	1.0 (ref)	1.13 (1.06, 1.21)	<0.001	1.0 (ref)	0.97 (0.90, 1.04)	0.357
Glucose (mmol/L)	1.0 (ref)	0.99 (0.98, 1.00)	0.109	1.0 (ref)	0.99 (0.98, 1.00)	0.154	1.0 (ref)	1.01 (0.99, 1.02)	0.287
HOMA-IR	1.0 (ref)	1.59 (1.48, 1.71)	<0.001	1.0 (ref)	1.49 (1.35, 1.64)	<0.001	1.0 (ref)	1.03 (0.94, 1.13)	0.538

The “random sample” comprises the 270 MHO individuals (from the full sample) plus 270 randomly selected MHNO individuals. The “matched sample” comprises the 270 MHO individuals plus 270 MHNO individuals who were matched, using propensity score matching, based on SBP, DBP, HDL, triglycerides, glucose, and HOMA-IR at baseline. HDL-C, triglycerides, glucose, and HOMA-IR were log-transformed due to skewed distributions; regression estimates were exponentiated and can be interpreted as ratios of geometric means

*BMI* body mass index, *DBP* diastolic blood pressure, *HDL-C* high-density lipoprotein cholesterol, *HOMA-IR* homeostatic model assessment of insulin resistance, *MHNO* metabolically healthy non-obese, *MHO* metabolically healthy obese, *SBP* systolic blood pressure, *WC* waist circumference

A total of 517 deaths, among the 4638 MHNO or MHO individuals, were observed over a median follow-up of 22.2 years (Table 5). In covariate-adjusted Cox proportional hazards models, the MHO group had a greater risk of mortality than the MHNO group in both the full sample (Hazard Ratio 1.57 (95% CI 1.15, 2.15)) and the random sample (2.11 (1.24, 3.58)). In the matched sample, however, there was less evidence of an association between MHO and all-cause mortality (1.34 (0.85, 2.13)).

**Table 5:** Differences in all-cause mortality between MHNO and MHO groups in the full sample, a 1:1 random sample, and a 1:1 matched sample, estimated using cox proportional hazards models

	*N* total	*N* (%) deaths	HR (95% CI)	*P*-value
*Full sample*				
MHNO	4368	472 (10.8)	1.0 (ref)	
MHO	270	45 (16.7)	1.57 (1.15, 2.15)	0.005
*1:1 random sample*				
MHNO	270	25 (9.3)	1.0 (ref)	0.006
MHO	270	45 (16.7)	2.11 (1.24, 3.58)	
*1:1 matched sample*				
MHNO	270	37 (13.7)	1.0 (ref)	
MHO	270	45 (16.7)	1.34 (0.85, 2.13)	0.209

The “random sample” comprises the 270 MHO individuals (from the full sample) plus 270 randomly selected MHNO individuals. The “matched sample” comprises the 270 MHO individuals plus 270 MHNO individuals who were matched, using propensity score matching, based on SBP, DBP, HDL-C, triglycerides, glucose, and HOMA-IR. The cox proportional hazards models were adjusted for sex, ethnicity, and baseline covariates (age, alcohol, smoking, mild and moderate exercise, occupational grade, GHQ, and diet)

*DBP* diastolic blood pressure, *GHQ* general health questionnaire, *HDL-C* high-density lipoprotein cholesterol, *HOMA-IR* homeostatic model assessment of insulin resistance, *MHNO* metabolically healthy non-obese, *MHO* metabolically healthy obese, *SBP* systolic blood pressure

## Discussion

The key finding of the present paper is that after accounting for baseline differences in cardio-metabolic disease risk factors, by matching the referent MHNO group to the MHO group on the risk factors used to define health status, the difference in mortality risk between the two groups was attenuated. As such, we provide evidence that documented associations of MHO with disease or mortality risk might be viewed as a statistical artifact that results from crudely dichotomizing continuous variables to define weight/health status. This finding does not mean that obesity is not deleterious when the cardio-metabolic disease risk factors we studied are at the same levels as those found in non-obese individuals, because our mortality analysis in the matched sample still revealed some residual risk associated with obesity (hazard ratio 1.34). This suggests that genuine healthy obese individuals are rare.

Much of the MHO literature has focused on prognosis, and numerous systematic reviews and meta-analyses have reported that MHO is associated with increased risk for various diseases (e.g., type 2 diabetes, cardiovascular events, chronic kidney disease, and depression) and mortality, compared to a healthy non-obese referent group [29–34]. Despite this strong evidence that MHO is not a benign condition, studies are still frequently published on this topic. For example, a recent analysis among 3.5 million adults found that MHO (compared to MHNO) was associated with a higher risk of developing coronary heart disease (Hazard Ratio 1.49 (95% CI 1.45, 1.54)), cerebrovascular disease (1.07 (1.04, 1.11)), and heart failure (1.96 (1.86, 2.06) over a mean follow-up of 5.4 years [13]. While many of these studies present baseline differences in cardio-metabolic disease risk factors between these two groups, which manifest from dichotomizing continuous variables, few discuss the impact of these baseline differences on the reported relationships.

To illustrate the fact that applying binary cutoffs to define weight/health status induces differences in cardio-metabolic disease risk factors between MHO vs MHNO groups (and between MUO vs MUNO groups) we also present 20-year trajectories. For HDL-C and triglycerides, higher average values among MHO than MHNO individuals remained remarkably similar in magnitude across follow-up, thereby suggesting that accounting for baseline differences (as in our mortality analyses) is approximately the same as accounting for cumulative differences over time. The differences for SBP and DBP reduced marginally over time, but those for glucose and HOMA-IR increased (e.g., from ~0.0 mmol/L at baseline to 0.2 mmol/L at 20-years for glucose). These findings are in agreement with previous Whitehall II study analyses showing that, relative to MHNO, the incidence of MHO individuals developing insulin resistance (incidence ratio 3.78 (95% CI 2.38, 5.99)) or high blood glucose (2.27 (1.43, 3.61)) over 20-years is higher than that for hypertension (1.35 (1.03, 1.77)) [5]. It appears that impairment of the glucose-insulin regulatory system might be the main factor driving transition to an unhealthy state, which would explain why the meta-analysed association of MHO with incident type 2 diabetes (relative risk ~4.0) is stronger than that for cardiovascular disease (relative risk ~1.2) [29, 32].

Importantly, our results do not mean that a person cannot be obese and have no complications. Key principles of normal variation mean that two obese individuals, even with exactly the same BMI, can (and most likely do) have different levels of cardio-metabolic disease risk factors. The idea that some people demonstrate some level of “resilience to obesity” is statistically plausible. And experimental studies in animal models [35, 36], in addition observational studies in humans [37], have started to reveal possible biological mechanisms (e.g., a genetic variant in humans near *ISR1* has been shown to be related to both increased percentage body fat and a favourable metabolic profile [38]) beyond the obvious (e.g., high BMI due to high fat-free mass). The problem is that MHO is a crude way of capturing heterogeneity in health among individuals with the same BMI level. For this reason, the concept of MHO may have limited clinically utility. In a meta-analysis of nearly 150,000 participants from 14 cohort studies, Lotta et al., for example, found that binary definitions of metabolic health only had satisfactory sensitivity (0.81 (95% CI 0.76, 0.86)) and low specificity (0.42 (0.35, 0.49) for predicting incident type 2 diabetes in obese individuals [33]. Despite these limitations, a large part of the field has not moved on from asking whether or not people can be obese yet healthy. In particular, we think that more research is needed on (1) the joint distributions of BMI and cardio-metabolic disease risk factors and (2) the life course exposures that might modify the relationship of BMI with incident disease or mortality. Such investigation would help us better understand the proportion and type of people who develop a high BMI without any adverse consequences.

The main strength of the present article is the thorough analysis of longitudinal data collected on a relatively large sample over 20 years of follow-up to address a novel research question. In terms of limitations, (1) the Whitehall II study sample is not representative of the wider UK population, although standard risk factor-cardiovascular disease associations in Whitehall II are comparable to those found in nationally representative studies [39], (2) we only used one definition of MHO, which does not incorporate other measures/indicators of adiposity (e.g., waist circumference), (3) the estimated relationships might be subject to residual confounding, and (4) there were not enough cases to investigate cause-specific mortality. While these types of considerations are important when trying to infer causality from observational data, we believe they are less important for our given research aim to demonstrate why other studies (which are subject to the same limitations) find what they find. The results we present are a demonstration of some of the possible consequences of converting continuous variables to binary concepts, and may be relevant to discussions on other related phenomena, such as the “fat but fit” paradigm [40].

## Conclusion

This paper demonstrates how dichotomising continuous variables results in different levels of cardio-metabolic disease risk factors at baseline and over 20 years of follow-up between MHO and MHNO individuals, despite both groups having the same label of “healthy”, and to a lesser extent between MUO and MUNO individuals. The greater disease and mortality risk of MHO compared to MHNO individuals, observed in large-scale epidemiological studies, is likely largely explained by the more deleterious risk factor trajectories (in the MHO group) that result from crude stratification. Future research needs to better quantify heterogeneity in disease and mortality risk among people with the same BMI, and investigate the characteristics and life-course factors that explain why some people develop a disease or die while other people with the same BMI do not.
